# Study on impact of robotic-assisted orthopaedic industrial noise (SIREN)

**DOI:** 10.1007/s00402-024-05303-8

**Published:** 2024-04-05

**Authors:** Joaquim Goffin, Emma MacRae, Luke Farrow, Duncan Whittaker, James Dixon, Iain Rankin, Anjan Krishnamurthy, Iain Stevenson

**Affiliations:** 1 Grampian Orthopaedics, Woodend Hospital, Eday Road, Aberdeen, AB15 6XS UK; 2 University of Aberdeen, Aberdeen, UK

**Keywords:** Hearing loss, NIHL, TKA, THA, Arthroplasty

## Abstract

**Introduction:**

The aim of this study was to evaluate noise exposure to the operating room staff consisting of the surgeon, assistant, anaesthetist and Mako Product Specialist (MPS) during Mako robotic-arm assisted total knee arthroplasty (TKA) and total hip arthroplasty (THA). We aimed to determine whether employees were exposed to noise at or above a lower exposure action value (LEAV) set out by the Noise at Work Regulations 2005, Health and Safety Executive (HSE), UK.

**Materials and methods:**

We prospectively recorded intra-operative noise levels in Mako robotic-arm assisted TKA and THA over a period of two months using the MicW i436 connected to an iOS device (Apple), using the Sound Level Meter App (iOS) by the National Institute for Occupation Safety and Health (NIOSH). Data obtained was then used to calculate “worst case” daily exposure value to assess if sound levels were compliant with UK guidelines. Comparison between operating room staff groups was performed with ANOVA testing.

**Results:**

A total of 19 TKA and 11 THA operations were recorded. During TKA, for the primary surgeon and the assistant, the equivalent continuous sound pressure level (L_Aeq_) was over 80 dB, exceeding the LEAV set out by the Noise at Work Regulations by HSE. During THA, the average L_Aeq_ and peak sound pressure levels did not exceed the LEAV. The calculated daily exposure for the primary surgeon in TKA was 82 dB. A Tukey post hoc test revealed that L_Aeq_ was statistically significantly lower in the anaesthetist and MPS (*p* < .001) compared to the primary surgeon and assistant in both TKA and THA.

**Conclusions:**

Operating room staff, particularly the primary surgeon and assistant are exposed to significant levels of noise during Mako robotic-arm assisted TKA and THA. Formal assessments should be performed to further assess the risk of noise induced hearing loss in robotic-arm assisted arthroplasty.

**Supplementary Information:**

The online version contains supplementary material available at 10.1007/s00402-024-05303-8.

## Introduction

Occupational work hazards such as the risk of surgical smoke inhalation, radiation exposure, and blood borne virus transmission are well recognised in orthopaedic surgery [1, 2]. More recently, attention has been drawn to the risk of noise-induced hearing loss (NIHL) in total knee arthroplasty (TKA) and total hip arthroplasty (THA) [3–8]. NIHL is an irreversible cause of sensorineural hearing loss which is associated with excessive exposure to recreational or occupational noise. Hearing loss is well-recognized in industries such as construction, manufacturing and mining but there is a paucity of evidence in the medical literature with regards to the risk of NIHL in orthopaedic surgery [9]. In Great Britain, there were an estimated 11,000 prevalent cases of work-related hearing problems between 2019 to 2022 [10].

The Health and Safety Executive (HSE) introduced The Control of Noise at Work Regulations (2005) for all industry sectors in the UK in 2006 [11]. This was introduced with a view to protect workers against the risk to their health from noise exposure at work. The regulations define actions based on Exposure Action Values, including lower exposure action values (LEAV) and upper exposure action values (UEAV) and the Exposure Limit Value (ELV) for personal daily or weekly noise exposure (Table 1). If the LEAV is reached or exceeded, employers should make a suitable and sufficient assessment of the risk from that noise to the health and safety of its employees. If UEAV are reached or exceeded, the employer has a duty to reduce noise at the source as reasonably practicable or provide hearing protection to its employees if unable to reduce the levels of noise. The area should be designated a Hearing Protection Zone and access should be restricted.

**Table 1:** HSE exposure limit values and action values

	Daily or Weekly personal noise exposure (A-weighted)	Peak sound pressure (C-weighted)
Lower exposure action values (LEAV)	80 dB	135 dB
Upper exposure action values (UEAV)	85 dB	137 dB
Exposure limit values	87 dB	140 dB

A recent study evaluating noise exposure during conventional hip and knee arthroplasty found that peak sound pressures during hip replacement surgery exceeded the exposure action values set out by the UK noise at work regulations. However, total knee arthroplasty did not exceed any exposure action values for peak sound pressure levels (L_Cpeak_) or daily personal noise exposure (L_EP, d_) [8].

The introduction of robotic-arm assisted surgery has gained popularity in recent years. Mako SmartRobotics™ (Stryker, Mahwah, NJ, USA) uses a combination of 3D CT reconstruction of the patient’s native anatomy, haptic technology for bone cuts and reaming, and insightful data analytics with an aim to improve surgeon reliability and reproducibility [12–15]. Limited evidence exists with regards to the potential for harmful sound levels in robotic-arm assisted surgery. One recent study has suggested that Mako TKA has higher continuous noise levels that other robotic systems, and may lead to NIHL in prolonged use [16]. They however did not explore noise exposure in Mako THA or assess the potential impact of cumulative exposure.

The aim of this study was therefore to evaluate the equivalent continuous A-weighted sound pressure level (L_Aeq_) and the peak sound pressure level (L_Cpeak_) exposure to the operating room staff consisting of the surgeon, assistant, anaesthetist and Mako Product Specialist (MPS) during Mako robotic-arm assisted primary TKA and THA. Using this data, we aimed to determine whether employees were likely to be exposed to noise at or above a lower exposure action value set out by the Noise at Work Regulations 2005, Health and Safety Executive (HSE), UK.

## Methods

Due to the nature of the study design ethical approval was not required. All participant information was anonymised. The study was registered with the local Qualitative Improvement and Assurance team; Project ID 5947. A prospective cohort study was performed to record intra-operative noise levels in Mako robotic-arm assisted (Stryker, Mahwah, NJ, USA) TKA and THA over a period of two months in a single centre. Data was collected for the operating surgeon, the surgical assistant, the anaesthetist and the MPS. Over the study period, 19 TKA and 11 THA procedures were recorded with full data. Only cases where complete data for all operating staff members was available were included. Cases involving partial knee arthroplasty, non-robotic or revision surgery were excluded.

The NIOSH Sound Level Meter application (iOS) by the National Institute for Occupation Safety and Health (NIOSH) for health-care professionals was used to collect noise exposure data. This application can record multiple metrics, including averages such as equivalent continuous A-weighted sound pressure levels (L_Aeq_) and time-weighted average sound levels (TWA), Max and Peak Levels (L_Cpeak_), Noise Dose and Projected Dose according to NIOSH and the Occupational Safety and Health Administration (OSHA) standard, and all three major weighting networks (A, C and Z). An external microphone, the MicW i436 (MicW Audio, Beijing, China), was connected to an iOS device (Apple) per NIOSH recommendations on the A-weight (dBA) scale. This microphone complies with the IEC 61,672 Class 2 sound level meter standard which means it is recognised as being able to measure sound levels accurately with regards to occupational noise exposure [17]. The Sound Level Meter application was calibrated using a RS PRO SLC 1356 Sound Level Meter Calibrator. This meets the International Standard IEC 60942:2003 class 2 and is designed for use with the class 2 sound level meters. It was used to calibrate the application and microphone at an output sound pressure level of 94 dB at a frequency of 1000 Hz at the start of each operating list.

Operations were recorded live with the use of the external microphone connected to an iPhone using an extension cable attached to the subject’s scrub at neck level using a collar clip, below the ear during the recorded procedures. Microphones were worn through each procedure by the operating surgeon, surgical assistant and MPS. The microphone was secured to a drip stand at the level of the patient’s head to replicate the distance of the anaesthetist from the noise source. Control noise levels were obtained by placing the recording device in the centre of the operating theatre and taking three, sixty second recordings. These recordings were taken with no other staff in the room and no background music. Anaesthetic equipment such as monitors and ventilation machines as well as laminar flow were left on during the control recordings.

All arthroplasty procedures were performed with set trays containing the same instruments. During TKA, femoral and tibial bone cuts were performed utilising either a standard or narrow oscillating Mako saw blade with a thickness of 2 mm, width 16 mm and thickness 2 mm and width 9 mm respectively. During THA, the saw blade used for femoral neck cuts was a Desoutter Stericut oscillating blade with a thickness of 1.27 mm, width 25 mm, useable length 90 mm and a tooth pitch of 2.1 mm. The broaches used for femoral preparation were the Exeter broaches, ranging from 30 to 44 mm offset sizes 0 to 4, 50 mm offset size 1 to 4, and 56 mm offset size 1 to 2. The acetabular reamers used were cutting edge acetabular reamers ranging from 36 mm to 74 mm in 1 mm increments.

To calculate the average sound levels of each operation, Leq A-weighted recordings (L_Aeq_) were taken from the moment the surgeon began scrubbing until the operation was over. The average noise recordings for both TKA and THA were used to calculate daily noise exposure.

For assessment of a worst-case scenario for noise exposure, we used an assumption that TKA and THA take 90 min on average to perform, with a typical operating day being 8 h. Data collated was input in the daily noise exposure calculator provided by HSE, which allows the user to determine the exposure points an employee accumulates for various tasks during a typical working day and provides reference values for an overall daily personal noise exposure (L_EP, d_). The lower Exposure Action Value (L_EP, d_ of 80 dB) and the Upper Exposure Action Value (L_EP, d_ of 85 dB) are represented respectively by 32 and 100 exposure points. To determine the exposure points and the daily noise exposure for each type of operation, the average noise level (L_Aeq_ dB) and exposure duration (hours) were inserted in the calculator provided by HSE.

### Statistical analysis

Statistical analysis was performed using IBM SPSS (version 28.0.1.1, Armonk, NY). Descriptive statistics were calculated with means ± standard deviation (SD) for all variables. Comparison between the four groups were performed with one way analysis of variance (ANOVA) testing followed by post-hoc Tukey pairwise tests. Normality of data was assessed using the Shapiro-Wilk test. Statistical significance was set at *p* < .05. To determine daily noise exposure, the noise exposure calculator provided by HSE was used.

## Results

### Control recordings

3 baseline sound recordings were obtained at the start of each operating list and the average was calculated. In the operating theatre where TKA recordings were obtained, L_Aeq_ was 65.4 dB, L_Cpeak_ was 94.7 dB. In the operating theatre where THA recordings were obtained, the average L_Aeq_ was 66.8 dB and L_Cpeak_ was 95.5 dB. 3 recordings of the saw with the microphone held at 50 cm were obtained with a duration of 30 s. The L_Aeq_ was 85.6 dB and L_Cpeak_ was 104.4 dB.

### Total knee arthroplasty

**Fig. 1:**
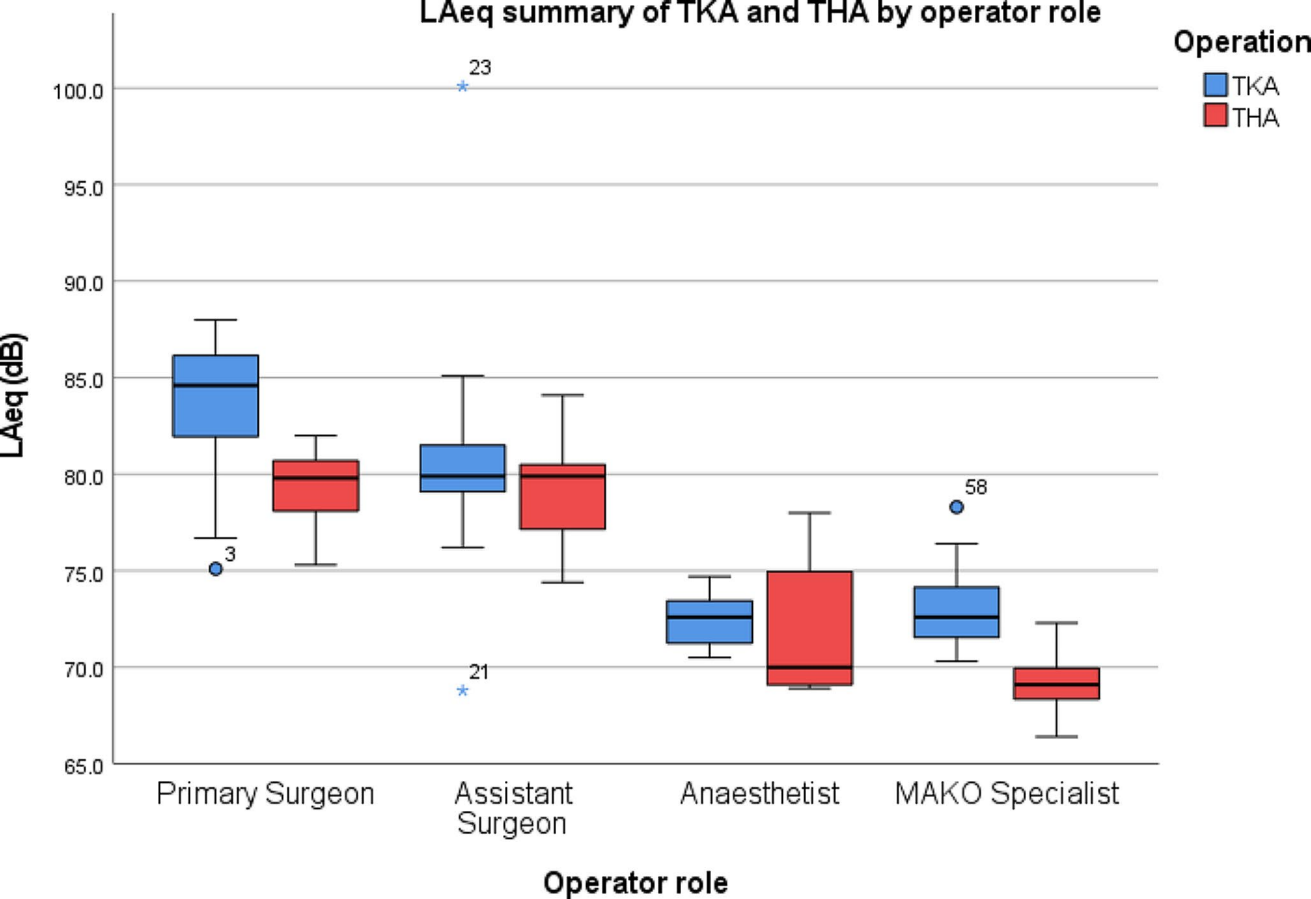
Summary of L_Aeq_ by operator role during TKA and THA. O = outlier, * = extreme outlier

**Fig. 2:**
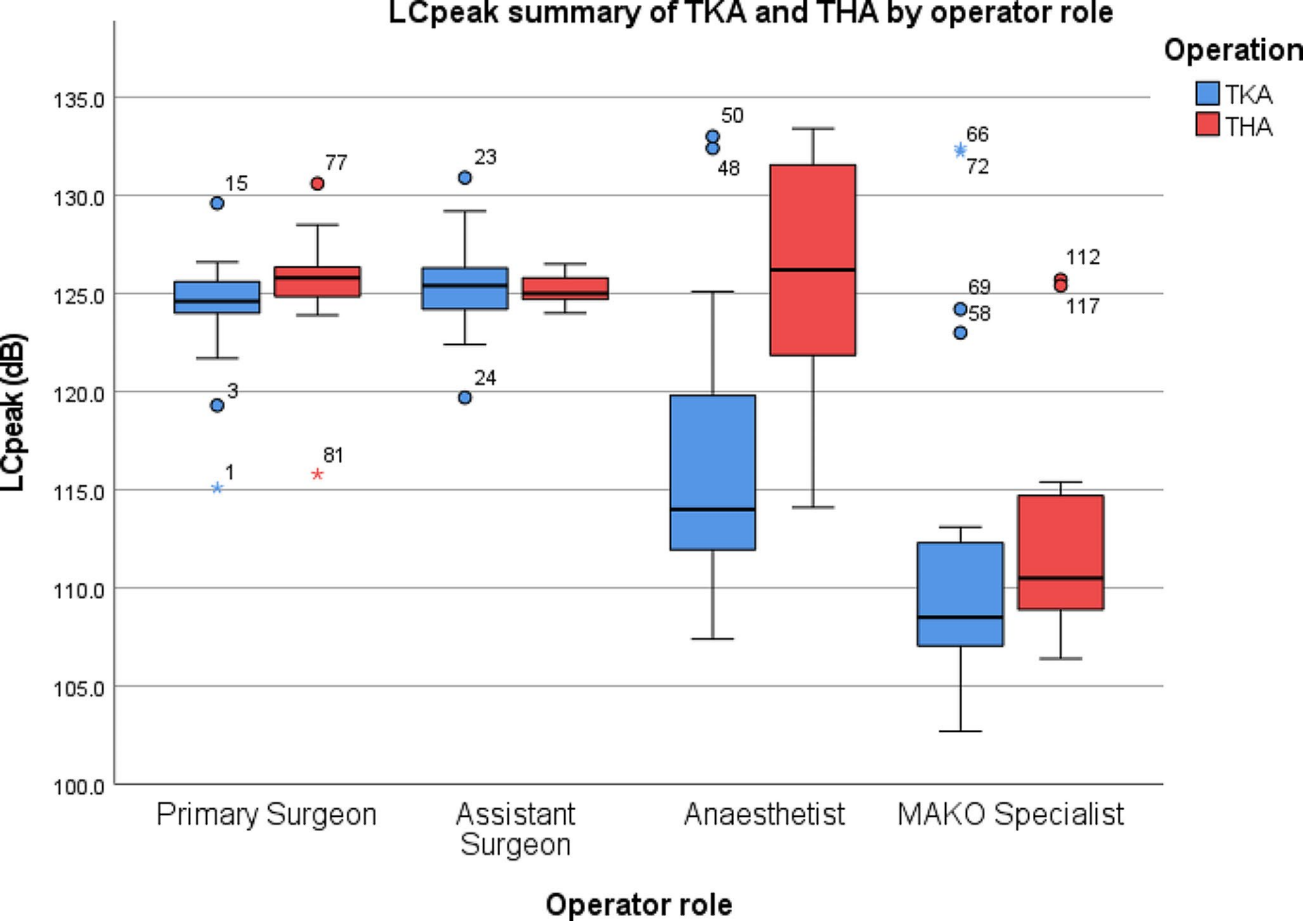
Summary of L_Cpeak_ by operator role during TKA and THA. O = outlier, * = extreme outlier

19 Mako TKA procedures were recorded in total. For the primary operating surgeon, the average L_Aeq_ was 83.7 dB and the L_Cpeak_ was 124.1 dB. For the assistant, the average L_Aeq_ was 80.8 dB and the LC_peak_ was 125.2 dB. For the anaesthetist, the average L_Aeq_ was 72.4 dB and the LC_peak_ was 116.4 dB. For the MPS, the average L_Aeq_ was 73.1 dB and the LC_peak_ was 112 dB (Table 2; Figs. 1 and 2).

**Table 2:** Summary of personal exposure for members of the operating theatre during TKA and THA. Superscript letters denote where differences between the primary surgeon and other groups were statistically significant (*p* < .05) on Tukey post hoc test. a = < 0.05

	Surgeon	Assistant	Anaesthetist	MPS	ANOVA results
TKA (*n* = 19)					
L_Aeq_ (dB(A)) ± SD (Range)	83.7 ± 3.4 (82–85.3)	80.8 ± 5.8 (78–83.6)	72.4 ± 1.4 (71.8–73.1) a	73.1 ± 2 (72.1–74) a	F (3,72). = 46.325, *p* < .001
L_Cpeak_ (dB(C)) ± SD (Range)	124.1 ± 3 (115.1–129.6)	125.2 ± 2.5 (119.7–130.9)	116.4 ± 7.2 (113–119.9) a	112 ± 9.1 (107.6–116.4) a	F (3,72) = 20.212, *P* < .001
THA (*n* = 11)					
L_Aeq_ (dB(A)) ± SD (Range)	79.2 ± 2.2 (75.3–82)	79.1 ± 2.7 (74.4–84.1)	72 ± 3.5 (68.9–78) a	69.1 ± 1.6 (66.4–72.3) a	F (3,40) = 42.897, *p* < .001
L_Cpeak_ (dB(C)) ± SD (Range)	125.3 ± 3.7 (115.8–130.6)	125.2 ± 0.8 (124–126.5)	125.4 ± 6.8 (114.1–133.4)	113 ± 6.7 (106.4–125.7) a	F (3,40) = 15.756 *P* < .001

There was a statistically significant difference between groups in terms of L_Aeq_ as determined by one-way ANOVA (*F*(3,72) = 46.325, *p* < .001). A Tukey post hoc test revealed that L_Aeq_ was statistically significantly lower in the anaesthetist (72.4 ± 1.4 dB, *p* < .001) and MPS (73.1 ± 2 dB, *p* < .001) groups compared to the primary surgeon (83.7 ± 3.4 dB) and assistant surgeon (80.8 ± 5.8 dB). There was no statistically significant difference in the L_Aeq_ between the primary surgeon and the assistant surgeon (*p* = .075).

There was a statistically significant difference between groups in terms of L_Cpeak_ as determined by one-way ANOVA (*F*3,72) = 20.212, *p* < .001). A Tukey post hoc test revealed that L_Cpeak_ was statistically significantly lower in the anaesthetist (116.4 ± 7.2 dB, *p* = .001) and MPS (112 ± 9.1 dB, *p* < .001) groups compared to the primary surgeon (124.1 ± 3 dB) and assistant surgeon (125.2 ± 2.5 dB). There was no statistically significant difference between the primary surgeon and the assistant surgeon (*p* = .941).

### Total hip arthroplasty

11 Mako THA procedures were recorded. For the primary operating surgeon, the average L_Aeq_ was 79.2 dB and the L_Cpeak_ was 125.3 dB. For the assistant, the average L_Aeq_ was 79.1 dB and the L_Cpeak_ was 125.2 dB. For the anaesthetist, the average L_Aeq_ was 72 dB and the L_Cpeak_ was 125.4 dB. For the MPS, the average L_Aeq_ was 74.8 dB and the L_Cpeak_ was 113 dB (Table 2; Figs. 1 and 2).

There was a statistically significant difference between groups in terms of L_Aeq_ as determined by one-way ANOVA (*F*(3,40) = 42.897, *p* < .001). A Tukey post hoc test revealed that L_Aeq_ was statistically significantly lower in the anaesthetist (72 ± 3.5 dB, *p* < .001) and MPS (69.1 ± 1.6 dB, *p* < .001) groups compared to the primary surgeon (79.2 ± 2.2 dB) and assistant surgeon (79.1 ± 2.7 dB). There was no statistically significant difference between the primary surgeon and the assistant surgeon (*p* = .999).

There was a statistically significant difference between groups in terms of L_Cpeak_ as determined by one-way ANOVA (*F*3,40) = 15.756, *p* < .001). A Tukey post hoc test revealed that L_Cpeak_ was statistically significantly lower in the MPS (113 ± 6.7 dB, *p* < .001) group compared to the primary surgeon (125.3.1 ± 3.7 dB), assistant surgeon (125.2 ± 0.8 dB) and drip stand (125.4 ± 6.8 dB). There was no statistically significant difference between the primary surgeon, the assistant surgeon and the anaesthetist (*p* = 1.000).

### Daily exposure

The daily noise exposure action value calculator was used to estimate the unprotected daily noise exposure of primary surgeons only, as the results demonstrated the highest recorded exposure for both TKA and THA procedures in this group. Based on a daily list consisting of four TKA procedures, with an average L_Aeq_ of 83.7 dB for the primary surgeon, the calculated daily exposure (L_EP, d_) was 82 dB, breaching the LEAV. Based on a daily list consisting of four THA, with an average L_Aeq_ of 79.2 dB for the primary surgeon, the L_EP, d_ was 78 dB, just below the daily lower exposure action value. A further summary of potential operating lists with a combination of both THA and TKA can be found in Table 3.

**Table 3:** “worst case” daily calculated noise exposure

Total duration	Description of activity	Exposure points per task	Daily noise exposure (L_EP, d_)
8 h	4 TKA	14	82
8 h	4 THA	5	78
8 h	3 THA & 1 TKA	5 (THA), 14 (TKA)	79
8 h	2 THA & 2 TKA	5 (THA), 14 (TKA)	81
8 h	1 THA & 3 TKA	5 (THA), 14 (TKA)	82

## Discussion

The most important finding of this study is that surgeons and assistant surgeons performing Mako robotic-arm assisted TKA are exposed to noise that exceed the lower exposure action values set out by HSE, according to the 2005 Control at Work Noise Regulations, with L_Aeq_ values of 83.7 dB and 80.8 dB respectively. Despite the average L_Aeq_ in THA not reaching or exceeding the LEAV over the 11 operations recorded, 36% of operations (4 of 11) for both primary surgeons and assistant surgeons reached or exceeded 80 dB(A). The L_Aeq_ for both groups was also under 1 dB below the LEAV, at 79.2 dB and 79.1 dB respectively. On average, L_Cpeak_ measurements of C-weighted peak pressures did not exceed the LEAV of 135 dB(C) for either TKA or THA. Subsequent hearing loss is therefore a potential consequence of Mako robotic-arm assisted surgical systems without the presence of auditory precautions.

During TKA, the estimated unprotected daily noise exposure (L_EP, d_) was 82 dB, which is above the LEAV set out by the HSE noise at work regulations. This likely relates to the prolonged duration of bone cuts in TKA, as well as the design of the saw itself [18]. This highlights the importance of regular, employer led noise risk assessment. In areas where the lower exposure action value is reached, employers should make personal hearing protectors available upon request. Employers should also provide employees with suitable and sufficient information, instruction and training. Palmer et al. found that conventional THA surgery routinely exposes surgeons to noise that exceeds exposure action values for peak sound pressure (L_Cpeak_) [8]. These high readings coincided with the insertion of the acetabular cup and preparation of the femoral canal. Although they found time-averaged daily noise exposure (L_EP, d_) to be within acceptable limits during THA, on one occasion this was recorded as 77.9 dB, suggesting that surgeons performing more than four THA would exceed the lower action values for L_EP, d_. Although our findings did not demonstrate that THA L_Cpeak_ reached the LEAV for any group, 36% of cases in the anaesthetist group exceeded 130 dB.

We previously conducted a study in our department assessing noise exposure during conventional TKA and THA using the same equipment and method described in this study [4]. The average L_Aeq_ for surgeons during TKA and THA was 80 dB and 77 dB respectively. This is lower than the values measured in this study. The L_EP, d_ for both conventional TKA and THA was also lower than the LEAV at 78 dB and 76 dB respectively. This reinforces the fact that whilst both conventional and robotic arm assisted arthroplasty are noise producing procedures which may exceed recommended safe levels as set out by HSE, MAKO robotic-arm assisted arthroplasty may produce more noise than conventional arthroplasty.

A systematic review by Mistry et al. analysed the evidence on the noise generated from orthopaedic operations and instruments and compared this with the 2003 European Commission Directive and 2005 UK HSE National Occupational Health guidelines [19]. As identified in our study, they concluded that safe levels of noise can be exceeded during orthopaedic operations. Slaven et al. also investigated noise exposure for TKA and THA and compared them with sound levels of arthroscopic procedures using a mobile phone based-app (NIOSH) connected to an iPhone [7]. They concluded that both operations have similar noise levels and neither exceeded the NIOSH guideline of 85 dB(A). They also found that peak pressure measurements (L_Cpeak_) were well below the NIOSH ceiling limit, with no cases exceeding the ceiling limit. One explanation for their findings is that the sound level meter was positioned in the breast pocket of the surgeon, with no mention of exact location of the microphone, or calibration of their device.

TKA demonstrated a significant difference in L_Aeq_ and L_Cpeak_ exposure between the primary and assistant surgeon groups compared to the anaesthetist and MPS groups on post-hoc analysis. THA demonstrated a significant difference in L_Aeq_ between primary and assistant surgeons compared to the anaesthetist and MAKO specialist. The primary surgeon, assistant surgeon and anaesthetist group demonstrated a significant difference in L_Cpeak_ exposure compared to the MAKO specialist. The difference in L_Aeq_ between groups can be explained by the distance of the groups from the noise source, with the primary surgeon and assistant surgeon being closest to the instruments used intra-operatively such as drills, mallets, reamers and saws. Evidence has been published on noise produced by powered instruments used in orthopaedic surgery, with levels exceeding 85 dB, which would explain the higher noise readings in members closest to the instruments [20, 21].

This is the first study to compare noise exposure for staff in the operating theatre using the MAKO robot for TKA and THA. From our findings, we have highlighted the potential risk of NIHL based on the UK Control of Noise at Work Regulations, provided by HSE. Hönecke et al., investigated noise exposure during robotic-assisted TKA [16]. They included three different robotic surgical devices including the MAKO robot (Stryker), NAVIO robot and CORI robot (Smith and Nephew). Their findings concluded that the L_Aeq_ was significantly higher during TKA using a MAKO robot (81.7 dB) compared to the sound levels of the NAVIO (77.4 dB) and CORI robot (77.9 dB). While the MAKO robot had the highest average sound level of the three robot systems, The NAVIO robot was found to have the highest peak sound levels. They concluded that robot-assisted TKA is a risk factor for NIHL. A possible explanation to the high L_Aeq_ readings could be attributed to the continuous noise generated by the ventilation of the robotic arm, even when not in use. Their findings with regards to the MAKO robot are comparable to what has been demonstrated in our results. This reinforces the accuracy of the results obtained and demonstrates the need for further formal assessments of noise exposure during primary TKA and THA, particularly with the increased use of robotic arm assisted arthroplasty. The results in this article demonstrate a health and safety consideration to the orthopaedic community. Further research is advised with regards to reducing the noise at source with the use of surgical instruments and power tools used in the operating theatre. Provision and use of protective earwear should be considered. Earplugs may negatively impact communication intra-operatively however, this may be overcome with the use of noise cancelling headphones.

A limiting factor for this study is the use of only one implant and robot for each TKA and THA. It may be that other implants or robotic devices produce different sound levels. We also recognise the small sample size in the study. This is in part due to the technical difficulty of ensuring that all iPhone devices were functioning correctly. During data collection, several phones cut off during recording, resulting in loss of data for individual members of the team. In this circumstance, all data was omitted from analysis as it prevented accurate between group analysis. The iOS app recordings did not identify the exact points where peak sound levels were recorded, such as when the robot or manual instruments such as hammers, saws and drills are used. Therefore, we were unable to identify key steps of each operation which may have contributed to breaching exposure action values. Further research assessing intra-operative sound levels with live graphs would enable us to further understand steps of the procedure where operating room staff may be most at risk of noise exposure exceeding exposure levels set out by HSE.

In conclusion, robotic arm assisted arthroplasty using the MAKO robot can exceed safe occupational levels of noise. Despite the existence of well-established HSE regulations, there is little evidence of formal assessments carried out in the workplace to assess the potential risk of noise exposure to staff as well as testing for NIHL in orthopaedic surgery. Operating room staff should be made aware of the risks associated with prolonged exposure to a noisy environment, particularly with the current growth in use of robotic assisted arthroplasty surgery.

### Electronic supplementary material

Below is the link to the electronic supplementary material.

Supplementary Material 1
