# Supplementary material for: Study on impact of robotic-assisted orthopaedic industrial noise (SIREN)
Source: Arch Orthop Trauma Surg. 2024 Apr 5;144(5):2413–20. doi: 10.1007/s00402-024-05303-8 (PMC11093793; doi:10.1007/s00402-024-05303-8)
Supplement: Supplementary file 1 — Supplementary Material 1 [file 402_2024_5303_MOESM1_ESM.docx]

**Compliance with Ethical Standards**

Paper title: Study on Impact of Robotic-assisted Orthopaedic Industrial Noise (SIREN)

We, the authors have approved the finals contents of the submission, been actively involved in the planning and enactment of the study, and have also assisted with the preparation of the submitted article.

We confirm that the article has not been submitted elsewhere, the references have been checked and are correct, and we have read the Submission Guidelines and the paper conforms to this Guide in all respects.

**1)** Conflicting interests: The authors declare no potential conflicts of interest with respect to the research, authorship, and/or publication of this article.

**2)** Funding: The author(s) received no financial support for the research, authorship, and/or publication of this article.

**3)** Ethical approval for this study was waived by the North of Scotland Ethics Service because this came under a service evaluation – designed and conducted solely to define or judge current care.

**4)** Informed consent declaration: not applicable

Joaquim Goffin

Specialty registrar

Grampian Orthopaedics

Woodend Hospital

Aberdeen

UK

E-mail: Joaquim.goffin3@nhs.scot
